# The role of cGAS-STING signaling in pulmonary fibrosis and its therapeutic potential

**DOI:** 10.3389/fimmu.2023.1273248

**Published:** 2023-10-25

**Authors:** Jing Zhang, Lanlan Zhang, Yutian Chen, Xiaobin Fang, Bo Li, Chunheng Mo

**Affiliations:** ^1^ Key Laboratory of Birth Defects and Related Diseases of Women and Children of MOE, State Key Laboratory of Biotherapy, West China Second University Hospital, Sichuan University, Chengdu, China; ^2^ School of Basic Medicine, Jining Medical University, Jining, Shandong, China; ^3^ State Key Laboratory of Respiratory Health and Multimorbidity, Department of Respiratory and Critical Care Medicine, West China Hospital, Sichuan University, Chengdu, China; ^4^ The Department of Endovascular Surgery, The First Affiliated Hospital of Zhengzhou University, Zhengzhou, China; ^5^ Fujian Provincial Key Laboratory of Critical Care Medicine, Department of Anesthesiology/Critical Care Medicine, Shengli Clinical Medical College of Fujian Medical University, Fujian Provincial Hospital, Fuzhou, China; ^6^ Department of Radiology, West China Hospital, Sichuan University, Chengdu, China

**Keywords:** pulmonary fibrosis, cGAS-STING, signaling pathway, inhibitors, therapeutic potential

## Abstract

Pulmonary fibrosis is a progressive and ultimately fatal lung disease, exhibiting the excessive production of extracellular matrix and aberrant activation of fibroblast. While Pirfenidone and Nintedanib are FDA-approved drugs that can slow down the progression of pulmonary fibrosis, they are unable to reverse the disease. Therefore, there is an urgent demand to develop more efficient therapeutic approaches for pulmonary fibrosis. The intracellular DNA sensor called cyclic guanosine monophosphate-adenosine monophosphate (cGAMP) synthase (cGAS) plays a crucial role in detecting DNA and generating cGAMP, a second messenger. Subsequently, cGAMP triggers the activation of stimulator of interferon genes (STING), initiating a signaling cascade that leads to the stimulation of type I interferons and other signaling molecules involved in immune responses. Recent studies have highlighted the involvement of aberrant activation of cGAS-STING contributes to fibrotic lung diseases. This review aims to provide a comprehensive summary of the current knowledge regarding the role of cGAS-STING pathway in pulmonary fibrosis. Moreover, we discuss the potential therapeutic implications of targeting the cGAS-STING pathway, including the utilization of inhibitors of cGAS and STING.

## 1 Introduction

Pulmonary fibrosis is a chronic and progressive lung disease that is a complex process involving many signaling pathways and the interaction of multiple cell types. It ultimately leads to destruction of lung structure and loss of gas exchange function, and even death (1, 2). Typically, bacterial infections, viral invasion, airborne irritants and pollutants will cause acute inflammation of the lungs, with fibrosis subsequently accompanying the inflammation (3, 4). Regardless of the mechanism that leads to pulmonary fibrosis, the end result is fibroblast proliferation and activation leading to large accumulation of extracellular matrix with concomitant inflammatory damage (5, 6). Pulmonary fibrosis as a progressive disease with interstitial lung disease (ILD) and idiopathic pulmonary fibrosis (IPF) being among the most lethal and irreversible forms of the disease progression (7, 8). The 5-year survival rate for IPF patients is 20% - 30%, with a median survival time of 2 to 3 years (9). With the increasing incidence of pulmonary fibrosis, the treatment situation is becoming more challenging.

Innate immunity is the first line of defense of the organism, and pattern recognition receptors (PRRs) are an essential component of the innate immune system, with diverse forms and wide distribution in cells. PRRs can recognize biomolecules with pathogen associated molecular pattern (PAMP) and damage associated molecular pattern (DAMP). PAMP includes double or single stranded DNA and RNA brought in by viral invasion or in the cytoplasm (10, 11). The cGAS protein, a member of PPR family, function as a DNA sensor. It was initially shown that cGAS recognizes exogenous DNA and triggers the innate immune system (12). Specifically, cGAS can detect exogenous DNA from bacteria, viruses, and protozoa in the cytoplasm (13). Activation of cGAS leads to the generation of 2′3′-cGAMP, which in turn induces phosphorylation of STING. This activation of the cGAS-STING innate immune pathway leads to the induction of type I interferon (IFN) gene expression (14). Type I interferons act in an autocrine manner, inducing expression of the STING gene. With increasing research into the cGAS-STING pathway, which is involved in autoimmunity, tumor immunity, cellular senescence, anti-viral and bacterial, this pathway may play a major role in the development of many disease (15–19).

Pirfenidone and nintedanib have been approved by the US Food and Drug Administration (FDA) for the treatment of pulmonary fibrosis (20). While these drugs have been shown to slow down the progression of the diseases, they are unable to reverse the disease. Lung transplantation, the last line of hope for patients, is not available to most patients due to its high cost and the risk of immune rejection (21). Research efforts are currently focused on investigating the molecular mechanisms that underlie the progression from acute lung inflammation to pulmonary fibrosis, as well as identifying new molecular pathways and therapeutic targets that can help prevent the development and progression of pulmonary fibrosis (4). Recent studies have highlighted the involvement of aberrant activation of cGAS-STING contributes to fibrotic lung diseases, such as sting-associated vasculopathy of infantile onset (SAVI), IPF, and silica-induced lung fibrosis (22, 23). Mutations in STING lead to SAVI, an autoinflammatory syndrome in children characterized by interstitial lung disease (24). Alveolar macrophage-derived exosomes carrying Ficolin B activate the cGAS-STING pathway, exacerbating lung injury and fibrosis (25). Additionally, the induction of cellular senescence through damaged autologous DNA-mediated cGAS activation contributes to lung fibrosis, while targeting the cGAS-STING pathway can bypass cellular senescence and attenuate the fibrotic process (26, 27). Furthermore, exposure to silica triggers cGAS-STING activation, resulting in lung inflammation and fibrosis (28–30). The present review offers a comprehensive summary of the current knowledge on the involvement of the cGAS-STING pathway in pulmonary fibrosis, as well as an exploration of cGAS and STING agonists and inhibitors, with a focus on their therapeutic potential for treating pulmonary fibrosis.

## 2 Discovery and history of cGAS-STING signaling

In 2008, Hiroki Ishikawa and Glen N. Barber discovered and first reported STING in antiviral immunity research. Concurrently, other research teams independently identified the protein and assigned it different names, including ERIS, MITA, and MPYS (31–33). STING was identified as a key adapter for induction of type I IFN during DNA virus infection or dsDNA transfection through the cDNA expression screening of luciferase reporter genes (31, 33, 34). How STING senses dsDNA was still unknown.

**Figure 1:**
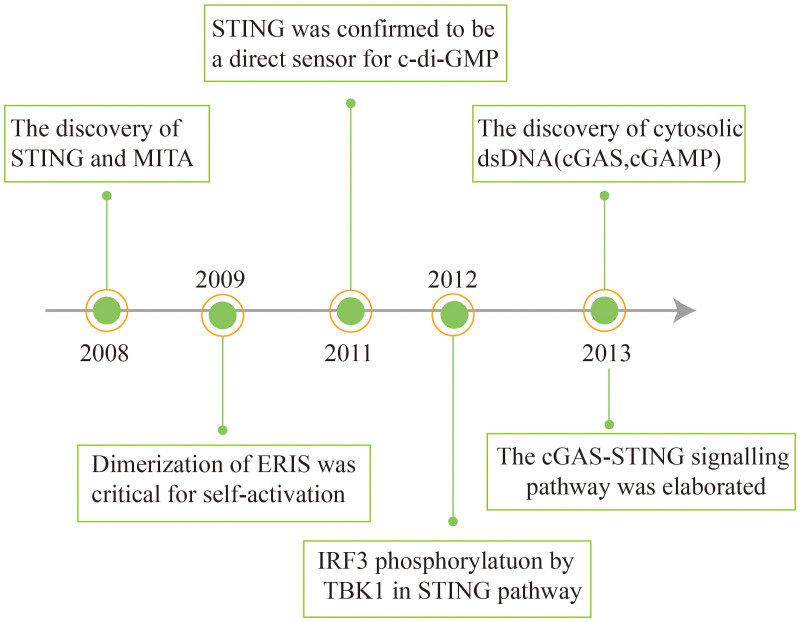
Schema outlining the history of interferon discovery and important events related to cGAS and STING biology.

Subsequently, it was found that STING recognizes cyclic diadenosine monophosphate (c-di-AMP) and cyclic diguanylatemo nophosphate (c-di-GMP) in bacteria (35, 36). cGAMP binds to STING as a second messenger (37). STING stimulates the phosphorylation of interferon regulatory factor 3 (IRF3) via the kinase TBK1 (TANK-binding kinase 1), thereby activating downstream signaling pathways (38). In 2013, the Chen group discovered that the host cell itself can synthesize cGAMP upon infection with pathogenic DNA, which activates the downstream STING. In another study, biotin-labelled DNA was used to affinity purify cGAS, an enzyme for the synthesis of cGAMP. It was found that cGAS directly recognizes exogenous DNA and catalyzes the conversion of ATP and GTP into cGAMP (39, 40). The cGAS-STING signaling pathway has thus been elaborated (**Figure 1**).

## 3 Overview of the cGAS-STING pathway

cGAS, a crucial enzyme involved in cytosolic DNA sensing, possesses a nucleotidyl transferase domain and two primary DNA binding domains (41). Activation of cGAS is primarily derived from invasive microorganisms such as DNA viruses, bacteria, or retroviruses, which introduce exogenous DNA into the cytoplasm, and from leakage of self-DNA from the nucleus or mitochondria, inducing its release into the cytoplasm (42, 43). Abnormally exposed dsDNA in the cytoplasm can be recognized by cGAS, which binds to the DNA and becomes a dimer. cGAS active site is altered to catalyze adenosine triphosphate (ATP) and guanosine triphosphate (GTP), ultimately resulting in the production of cGAMP (44, 45). The cGAMP is then detected by the ER membrane protein STING, which exists as a dimer with two cytoplasmic domains (31, 46).

**Figure 2:**
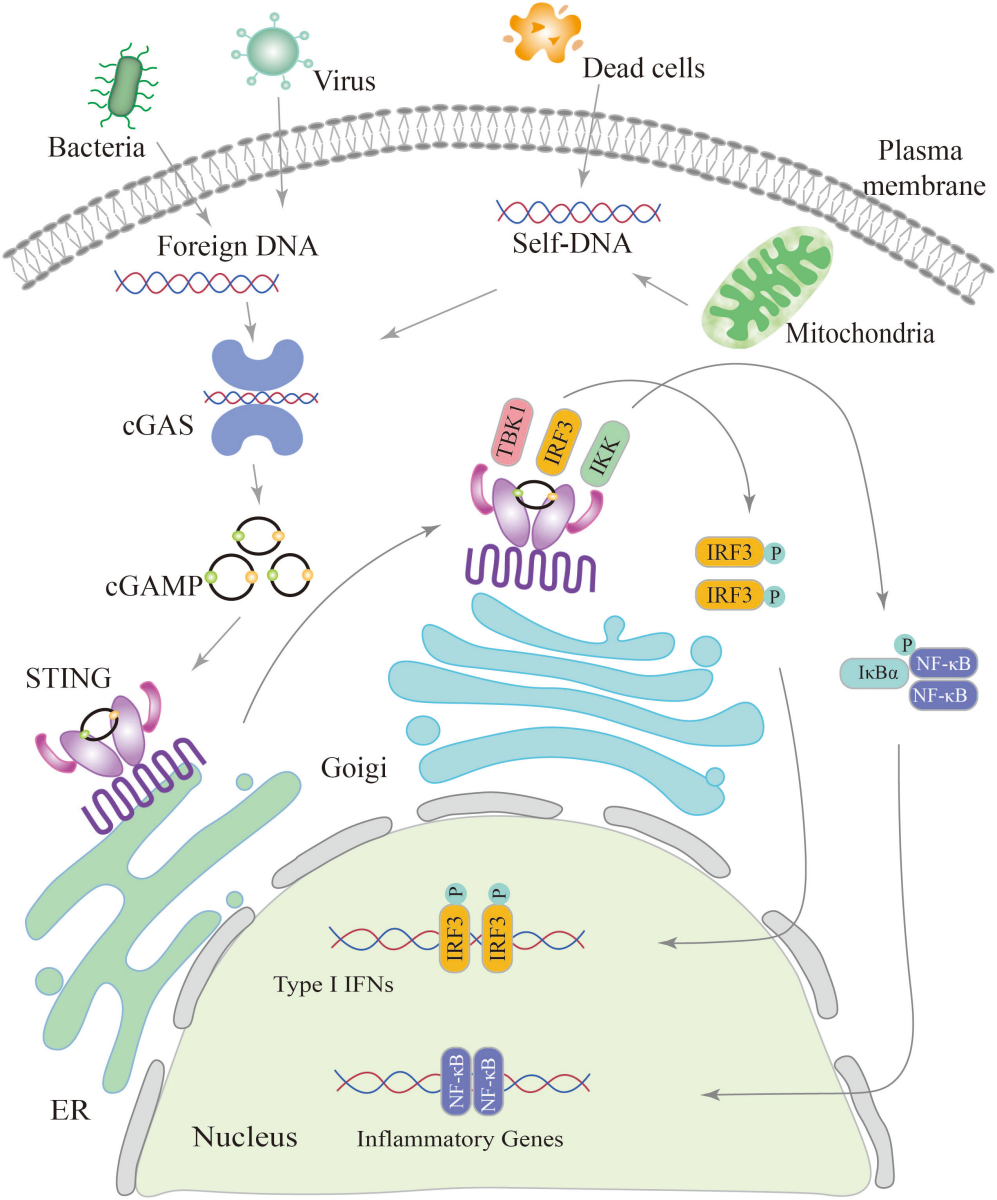
Schematic diagram of the cGAS-STING signaling pathway. The cGAS-STING pathway is initiated by the detection of cytosolic DNA, including nucleic acids from pathogens and self-DNA. When cytosolic DNA is recognized, cGAS binds to STING, leading to the translocation of STING from the endoplasmic reticulum (ER) to the Golgi and post-Golgi compartments. This activation of STING triggers the phosphorylation of IRF3 by TBK1, resulting in gene expression of type I interferons. Additionally, STING activates NF-κB through the phosphorylation of the kinase IKK, leading to the activation of genes involved in inflammation and immune responses.

When 2′3′-cGAMP binding to STING, it triggers a conformational change in the protein, leading to its oligomerization (47, 48). STING oligomers are transported from the endoplasmic reticulum to the Golgi apparatus (31, 49, 50). Within the Golgi compartment, STING facilitates the recruitment and activation of TBK1. Recruited TBK1 directly phosphorylates the carboxyl terminus of STING, creating a dedicated docking site for IRF3 (51). After being phosphorylated and activated by TBK1, IRF3 initiates downstream signaling pathways (52). Following phosphorylation, IRF3 undergoes dimerization and translocates to the nucleus, driving the production and secretion of a variety of cytokines including type I IFNs (52, 53). STING triggers the activation of IκB kinase (IKK), leading to the phosphorylation of the IκB repressor family that regulates the NF-kB. NF-kB is then translocated to the nucleus, leading to induce gene expression of cytokines such as interleukin 6 (IL-6) and tumor necrosis factor-α (TNF-α) (54, 55) (**Figure 2**).

## 4 Overview of cGAS and STING antagonists

In most lung diseases characterized by increased inflammation and elevated levels of pro-inflammatory factors, inhibition of the cGAS-STING signaling provides an avenue for therapeutic intervention. Therefore, cGAS-STING signaling inhibitors may be developed for various interferon-related diseases (**Table 1**).

**Table 1:** cGAS and STING antagonists.

cGAS and STING antagonists		References
*cGAS Catalytic site inhibitors*	PF-06928125	(56)
	RU.521	(57)
	G150	(58)
	Compound S3	(59)
*Disrupt DNA binding*	X6	(60)
	Suramin	(61)
	A151	(62)
	Hydroxychloroquine	(63)
	Quinacrine	(63)
*Targeting the CDN-binding site*	Tetrahydroisoquinolines (compound 18)	(64)
	Astin C	(65)
	SN-011	(66)
*Targeting STING palmitoylation sites*	Nitrofurans (C-176 and C-178)	(67)
	Indole ureas (H-151)	(67)
	Nitro fatty acids (NO2-FA)	(68)
	Acrylamides (BPK-21, BPK-25)	(69)
*unknown mechanism*	CU-76	(70)
	compound 13	(71)
	SP23	(72)
	VS-X4	(73)

Overview of cGAS and STING agonists is presented in **Supplementary**.

### 4.1 cGAS catalytic site inhibitors

Numerous cGAS antagonists have been identified through studies of the cGAS crystal structure, which bind to the active site. This has led to the development of several cGAS inhibitors that compete with ATP or GTP substrates, as well as the product cGAMP. One example is PF-06928125, which binds to the cGAS nucleotide binding site and reduces its activity by disrupting cGAMP binding (56). Similarly, compound S3 has also shown the ability to act as a cGAS inhibitor (59). Moreover, RU.521 has the capability to bind to the active site of cGAS and diminish its binding affinity for ATP and GTP, which is promising as an inhibitor of cGAS in mouse studies. One of the best h-cGAS inhibitors, G150, specifically targets the ATP and GTP binding pockets of cGAS, influencing the formation of the second messenger 2’,3’-cGAMP (57, 58).

### 4.2 Disrupt DNA binding

The main principle of the other major class of cGAS antagonists involves competing with cGAS for DNA binding. A series of experiments have shown that antimalarials compounds, such as A151, X6, hydroxychloroquine and quinacrine, as well as sulforaphane, can inhibit the perception of dsDNA by the DNA receptor cGAS. These compounds are believed to achieve this inhibition by binding to the dsDNA binding site of cGAS. This binding disrupts the formation of the cGAS-dsDNA, thereby inhibiting cGAS activation (60–63).

### 4.3 Targeting the CDN-binding site

The binding of cyclic dinucleotide (CDN) to STING is essential for the activation of downstream signaling pathways. However, disrupting this binding can act as a competitive antagonist of STING. Certain compounds, such as tetrahydroisoquinoline analogue compound 18 and plant-derived cyclic peptide astin C have been shown to competitively bind to the CDN site of STING. By binding to STING, these compound prevent the biding of cGAMP and subsequently inhibits downstream signaling (64, 65). Another STING antagonist, known as SN-011, processes a greater affinity for the CDN binding pocket of STING compared to the naturally 2’3’-cGAMP. SN-011 effectively locks STING in an open inactive conformation, thereby inhibiting downstream production of IFN and inflammatory cytokine (66).

### 4.4 Targeting STING palmitoylation sites

The second class of inhibitors target specific cysteine residues, such as Cys88 or Cys9, located near the transmembrane structural domain of STING proteins. These cysteine residues are affected by palmitoylation. Ablasser’s lab found that nitrofuran derivatives, including C-170, C-171, C-176, C-178, and H-151, can block the STING-mediated signaling pathway. These compounds exert their inhibitory effects by chemically modifying the Cys91 residue within STING and inhibiting STING palmitoylation (67). NO2-FAs hinder STING palmitoylation through s-nitroalkylation reactions. Specifically, they inhibited STING activation by nitroalkylation of Cys88 and Cys99 mercaptans located in the amino -terminal region of STING (68). Cys91 in STING has been shown to be palmitoylated and targeted by the covalent ligand BPK-25, which inhibits STING activation via disrupting the binding of the cyclic dinucleotide ligand cGAMP (69).

### 4.5 Inhibitors of unknown mechanisms

SP23 is a STING protein degrader designed for proteolysis-targeting chimera (PROTAC) technology based on a small molecule STING inhibitor (C-170) and pomalidomide (72). There are also several inhibitors which mechanisms are still unknown. For example, cU-76 has been found to bind to cGAS, but does not disrupt the binding between cGAS and dsDNA. The exact mechanism by which cU-76 binds to cGAS and inhibits its function is still unclear and requires further investigation (70). Similarly, the small heterocyclic compound VS-X4 inhibits STING, but the exact mechanism by which it exerts its inhibitory effects is not clear. Additionally, compound 13 has been shown to regulate the cGAS-STING pathway, but its precise mechanism of action has not been elucidated (71, 73). Further studies are required to uncover the specific mechanisms of action for these inhibitors.

## 5 Innate immune parameters specifically affected by the cGAS-STING pathway during pulmonary fibrosis

**Figure 3:**
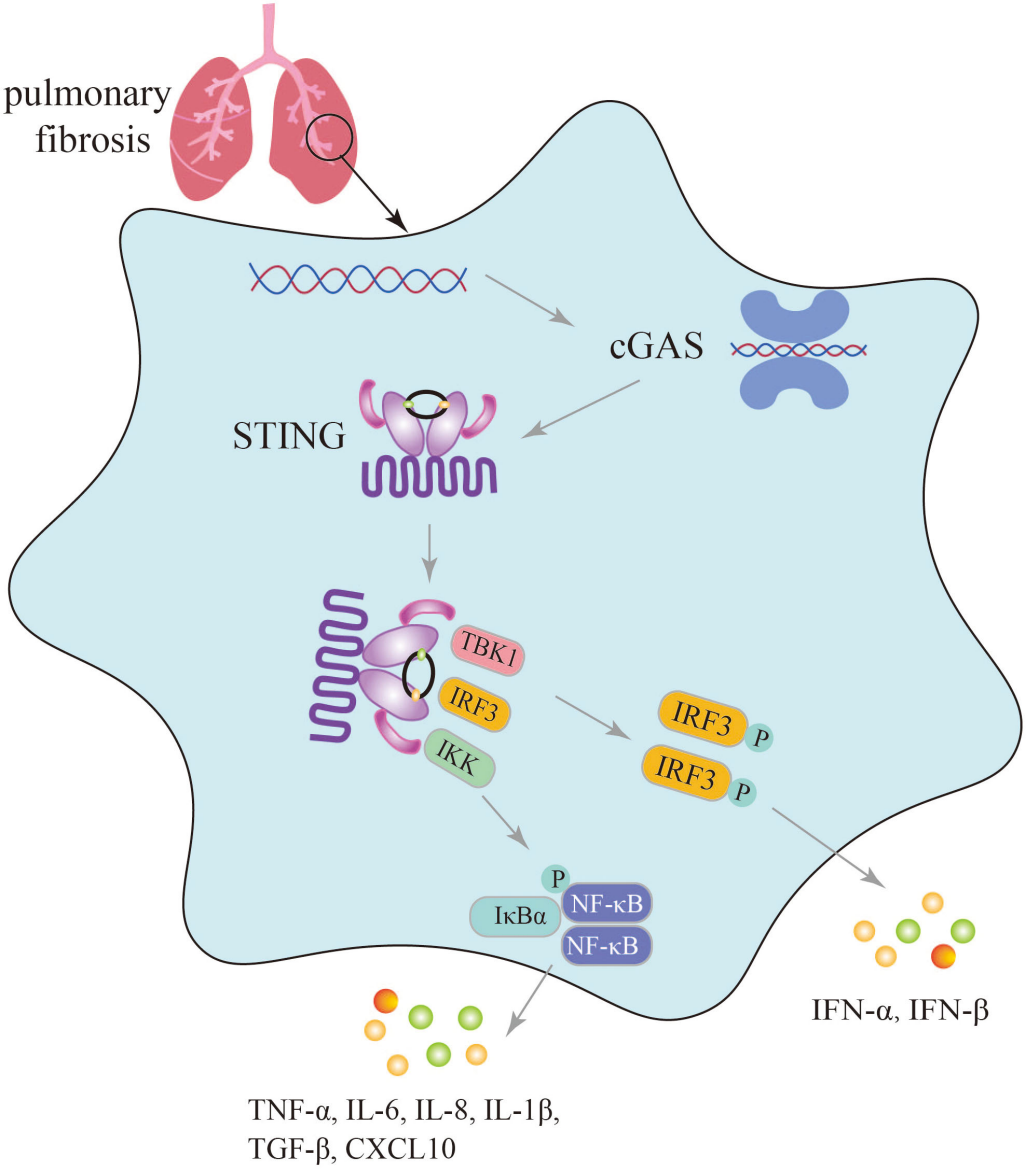
Secretion of immune inflammatory factors, such as TNF-α, IFN-β induced by the cGAS-STING pathway in the progression of pulmonary fibrosis.

cGAS, a member of the PRR family, recognizes endogenous and exogenous DNA, activates innate immunity, and affects numerous autoimmune diseases such as SAVI, Systemic lupus erythematosus (SLE), and Aicardi-Goutières syndrome (AGS) (19). Furthermore, an increasing body of research has indicated that aberrant activation of the cGAS-STING pathway can lead to excessive and sustained production of inflammatory factors, including type I IFNs, contributing to the development of various diseases (74). The undeniable role of the cGAS-STING signaling pathway in innate immune diseases is supported by Al Khatib et al., who demonstrated that TOP1MT influences the activation of the mtDNA-mediated cGAS-STING innate immune response, and that the P193L variant may contribute to the autoimmune phenotype observed in patients (75). The inflammatory response induced by activation of the cGAS-STING signaling pathway is a significant factor in the progression of pulmonary fibrosis. Conversely, blockage of cGAS-STING signaling pathway to suppress inflammatory response is an important aspect of lung fibrosis treatment (**Figure 3**).

In the lungs exposed to silica, which triggers dsDNA production, activation of the cGAS-STING signaling pathway induces lung inflammation and fibrosis, with significantly elevated levels of TNF-α, IL-6, TGF-β, and IFN-β, as well as increased levels of CXCL10 in the sputum of silicosis patients (28, 30). In mice with graphitized multi-walled carbon nanotube (GMWCNT)-induced pulmonary fibrosis, high expression of cGAS, STING, IFN-β, NF-κB, IL-1β, and TGF-β1 indicates the induction of an inflammatory response through activation of the cGAS-STING signaling pathway (29). In bleomycin-induced pulmonary fibrosis, activation of the cGAS-STING signaling pathway not only leads to the production of numerous SASP factors such as IL-6, IL-8, and the upregulation of cell cycle-related factors p16 and p21, but also increased secretion of inflammatory factors like IFN-β (26, 27).

In the treatment of pulmonary fibrosis, an increasing number of natural Chinese medicines are demonstrating significant efficacy. For example, Tanreqing and Juglanin exhibit potent anti-inflammatory and antioxidant effects. They not only inhibit the expression of fibrosis-related genes but also effectively suppress the expression of inflammatory factors such as IL-6, TNF-α, and IL-1β by inhibiting the cGAS-STING signaling pathway, thereby alleviating fibrosis (76, 77).

## 6 Targeting cGAS-STING signaling alleviates pulmonary fibrosis

PRRs located on the cell surface or within different cells are essential components of innate immunity. They possess the ability to accurately recognize PAMPs, triggering an inflammatory response and activating the immune system to defend against invasion by pathogenic microorganisms (10). cGAS, along with Toll-like receptors (TLRs) and RIG-I-like receptors (RLRs), belongs to the PRR family. TLRs primarily recognize extracellular pathogen-related substances or extracellular nucleic acids that are phagocytosed by phagocytes (10). RLRs are primarily responsible for detecting intracellular RNA (78). TLR3 has been reported to the regulation of lung fibrosis by recognizing viral dsRNA (79). Recent studies have shown that cGAS-STING primarily participates in the development and progression of pulmonary fibrosis by responding to both exogenous and endogenous DNA. Targeting cGAS-STING signaling has shown potential in alleviating pulmonary fibrosis (30, 80).

### 6.1 Gain-of-function mutation in STING leads to pulmonary fibrosis

**Figure 4:**
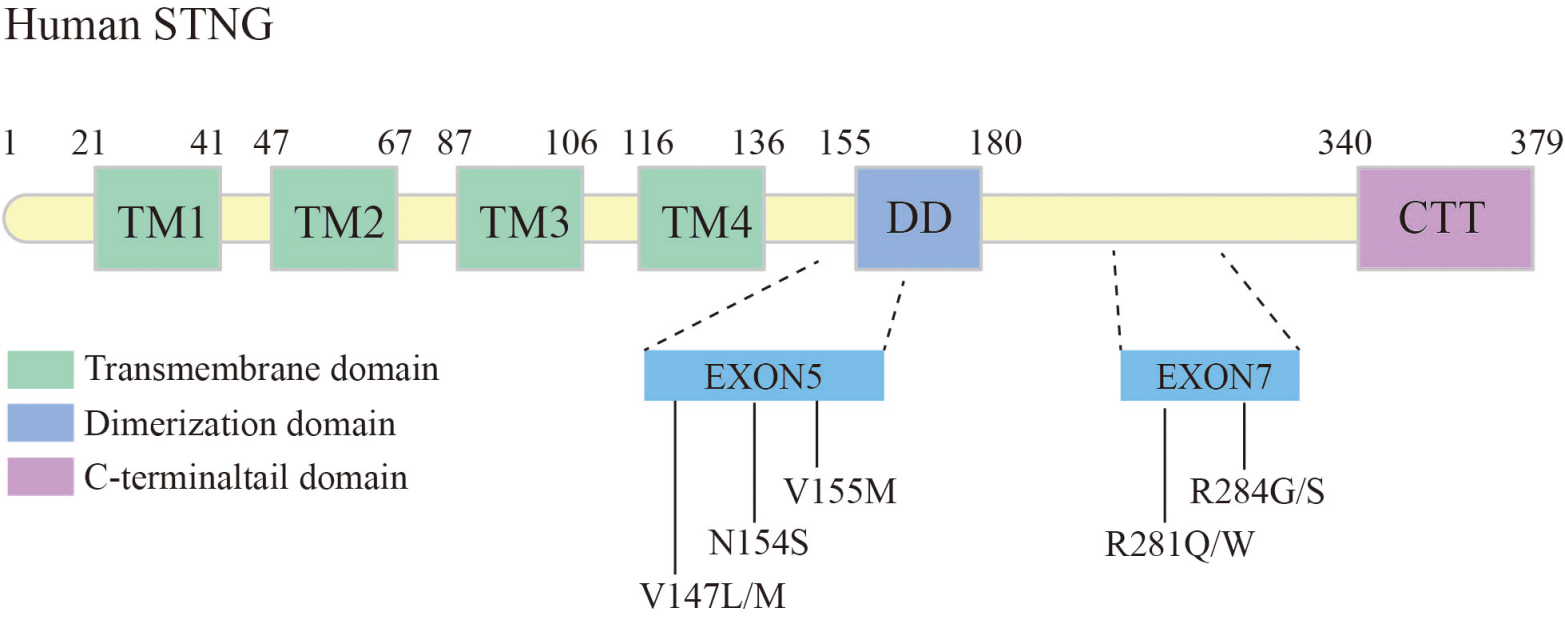
Schematic structure of human STING, and mutation sites leading to pulmonary fibrosis.

Gain-of-function mutations in the STING-encoding gene, TMEM173, have been shown associated with pulmonary fibrosis. Liu et al. elaborated six patients with STING-associated vasculopathy of infancy (SAVI), caused by heterozygous *de novo* gain-of-function mutations in the STING. These cases exhibited systemic inflammatory responses in the neonatal period, severe cutaneous vasculopathy resulting in tissue necrotic loss, interstitial lung disease (ILD), and the pathological features of pulmonary fibrosis (24) (**Figure 4**).

Among the identified mutations in STING, the V155M is the most common mutation locus. This mutation can lead to autoimmune diseases such as malignant rashes and systemic lupus erythematosus, while a proportion of patients have concomitant lung inflammation and fibrosis (24, 81). Other mutations occurring in exon 5, such as N154S, V147L, and V147M, have also been associated with lung inflammation and fibrosis (24). The structure model of STING revealed the structure changes associated with Class I variants, including N154S, V155M, V147L, and V147M. Theses variants have been shown to control the 180° rotation of the ligand-binding region in the STING dimer induced by cGAMP ligand, thereby inducing polymerization and activation of STING (48).

Studies have reported variant R284S/G and R281Q/W in exon 7 of STING causing SAVI, some patients also suffered from severe interstitial lung disease (82–84). Konno et al. found that the AMPK inhibitor GSK 690693 has a potent ability to inhibit STING (R284S)-mediated gene induction, mainly by inhibiting STING-controlled IRF3 phosphorylation (85). Some reports have reported an overall improvement in SAVI with JAK1/2 inhibitors (ruxolitinib or baracitinib) (86, 87). SAVI patients treated with ruxolitinib have been found to reduced symptoms after follow-ups (88). However, the mechanisms through which single-gene defects in the STING pathway contribute to lung fibrosis are still not fully understood. Further research is necessary to gain a better understanding of these mechanisms, which will contribute to improved patient management in the future.

### 6.2 Natural products attenuate pulmonary fibrosis by targeting STING signaling

Panax ginseng Meyer is an important traditional Chinese medicinal herb. Ginsenosides, which are the active compounds in ginseng, are triterpenoid saponins that include 20(S)-Protopanaxadiol (PPD) (89, 90). These compounds possess pharmacological properties such as antiviral, antioxidant, and immunomodulatory activities, and have the potential to be used as therapeutic agents for treating organ fibrosis. Recent studies have shown that ginsenosides can alleviate pulmonary fibrosis and reduce lung injury (91, 92). In a mouse model of bleomycin-induced lung fibrosis, Ren et al. found that PPD extracted from ginseng attenuated lung fibrosis induced by bleomycin, as evidenced by a higher survival rate and lower levels of fibrotic markers such as a-SMA, TGF-β1, and collagen I (93). Mechanistically, intraperitoneal injection of PPD suppressed STING signaling through activation of AMPK in mouse alveolar epithelial cells. Furthermore, the anti-fibrotic effect of PPD was blocked by combined injection of the STING activator CMA or the AMPK inhibitor Compound C. In contrast, injection of the STING inhibitor C-176 or the AMPK activator metformin mimicked the therapeutic effect of PPD. These findings suggest that the ginseng saponin metabolite PPD alleviates lung fibrosis by regulating AMPK and STING signaling (93). The therapeutic effect of PPD in patients with idiopathic pulmonary fibrosis (IPF) awaits further clinical investigation.

Tanreqing injection (TRQ) is a widely recognized traditional Chinese patent medicine used for treating the syndrome of phlegm-heat obstructing the lungs by resolving phlegm, clearing heat, and relieving cough (94). TRQ is also employed for various pulmonary diseases, including IPF. Deng et al. found that intraperitoneal injection of TRQ can reduce the infiltration of inflammatory cells, expression and secretion of inflammatory cytokines (TNF-α, IL-6, and IL-1β), and lung fibrosis induced by bleomycin administration (77). The fibrotic lungs showed elevated levels of STING, which were inhibited by TRQ injection, suggesting that TRQ exerts its therapeutic effect by blocking the STING pathway. Clinical studies have demonstrated that TRQ can reduce the progression of pulmonary fibrosis and enhance lung function (77). TRQ is composed of five traditional Chinese medicines, including Selenaretos thibetanus Cuvier (known as Xiongdanfen in Chinese, making up 3.8%), Scutellaria baicalensis Georgi (referred to as Huangqin in Chinese, making up 23.6%), Capra hircus Linnaeus (known as Shanyangjiao in Chinese, making up 1.9%), Lonicera japonica Thunb (referred to as Jinyinhua in Chinese, making up 23.6%), and Forsythia suspensa (Thunb.) Vahl (known as Lianqiao in Chinese, making up 47.1%) (95). Further experiments are needed to identify the specific components of TRQ that regulate the STING pathway and inhibit lung fibrosis. Additionally, the molecular and cellular mechanisms by which TRQ regulates STING signaling require further investigation.

Radix Pseudostellariae (RP), commonly known as Tai Zi Shen, is a type of tonic herbs used in traditional Chinese medicine. It is well-regarded for its notable pharmacological effects, including antioxidant properties, hypoglycemia effects, anti-fatigue benefits, and memory improvement (96). Heterophyllin B, a occurring cyclic peptide naturally purified from Radix Pseudostellariae, exhibits anti-inflammatory and antioxidant properties (97, 98). Researches have showed that heterophyllin B can reduce myofibroblast recruitment and ECM deposition via blocking the AMPK-STING pathway. *In vitro* studies using MLE-12 cells incubated with TGF-β1 have shown that heterophyllin B effectively reduce STING production and activating AMPK. In animal models, heterophyllin B exerted its anti-lung fibrosis effect in mice by promoting AMPK, reducing STING overexpression and activation, and inhibiting bleomycin-induced energy metabolism abnormalities in the lung (99).

**Figure 5:**
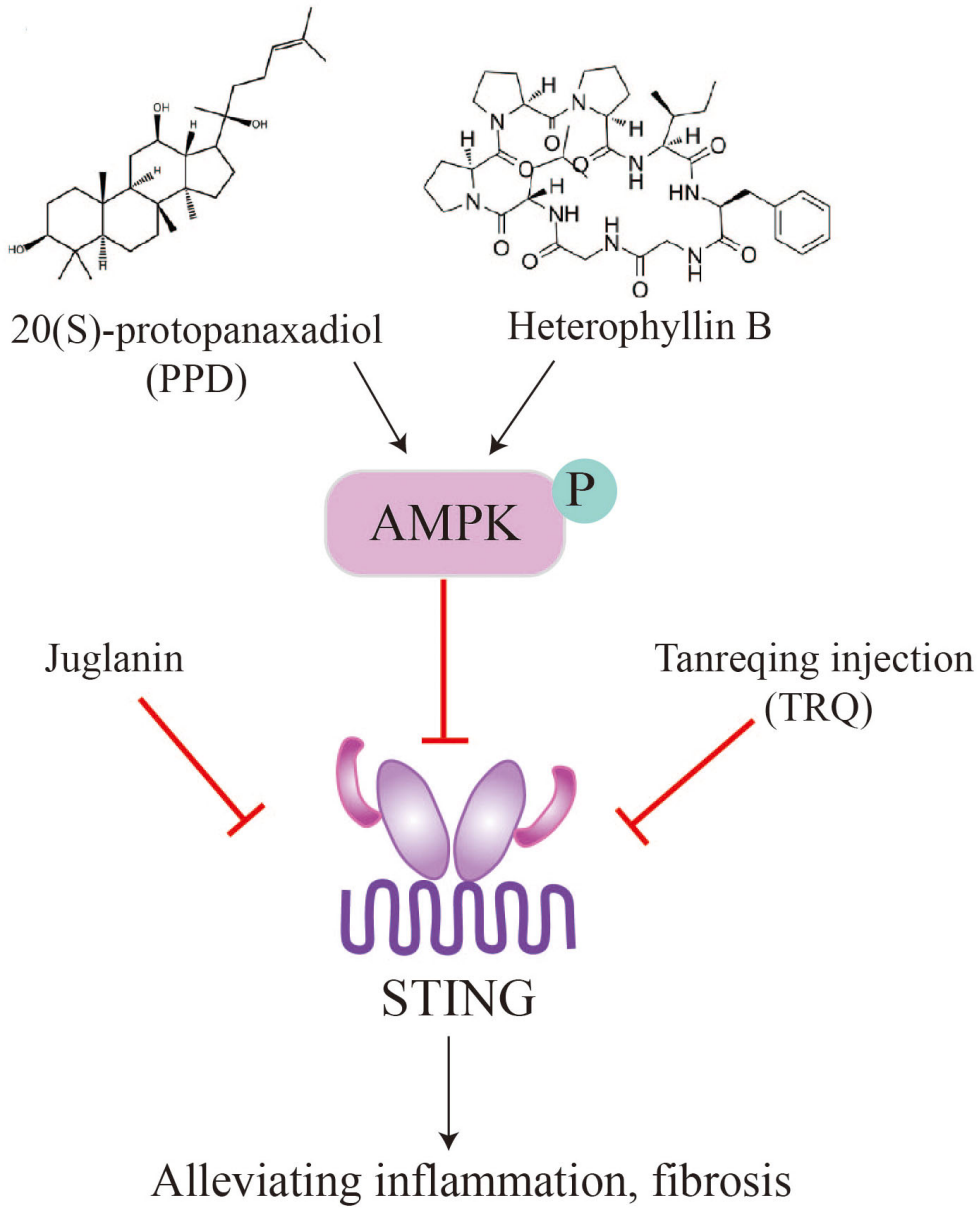
Natural products, such as PPD, heterophyllin B, juglanin, TRQ, alleviate pulmonary fibrosis by inhibiting STING.

Juglanin is a flavonoid glycoside isolated from the Chinese herb He Shou Wu. It has been found to possess anti-inflammatory, anticancer effects, and antioxidant (76, 100, 101). In the bleomycin-induced lung fibrosis model, Juglanin treatment significantly downregulated fibrosis markers, such as matrix metalloproteinase 9, TGF-β1, fibronectin, α-smooth muscle actin (α-SMA), and collagen type I. This resulted in the attenuation of lung fibrosis and a reduction in inflammation. The mechanism of action appears to involve the blockage of STING signaling (80). These findings suggest that natural products can alleviate pulmonary fibrosis and inflammation by suppressing STING, providing potential avenues for future drug development and fibrosis treatment strategies (**Figure 5**).

### 6.3 Targeting the cGAS-STING pathway bypasses cellular senescence to attenuate pulmonary fibrosis

Cellular senescence is a state of permanent cell growth arrest characterized by alterations in cell morphology and physiological metabolism. These changes result in the secretion of various pro-inflammatory cytokines and chemokines, as well as vascular growth factors, collectively known as the senescence-associated secretory phenotype (SASP) (102, 103). The excessive production of pro-inflammatory molecules during cellular senescence can lead to chronic inflammation and impaired tissues regeneration, resembling the process of progressive fibrosis. There is increasing evidence that key cells involved in lung fibrosis, such as alveolar type 2 progenitor cells (AT2) and pulmonary fibroblasts, undergo a senescence process during pathological lung hyperplasia (104, 105). Senescence can be induced by various factors, including DNA damage, telomere dysfunction, mitochondrial dysfunction, and oncogene activation (106). Studies have also shown that damaged DNA binding to the DNA sensor cGAS can induce cellular senescence (107, 108).

In pulmonary fibrosis, leakage of damaged mtDNA or self-DNA into the cytoplasm activates cytoplasmic cGAS, causing an inflammatory response that triggers cellular senescence. Recent findings on IPF-LFs (lung fibroblasts) and IPF-AECs (alveolar epithelial cells) has also implicated mtDNA and cGAS as potential mediators of senescence. Targeting cGAS pharmacologically (using inhibitor RU.521) or genetically (siRNA transfection) can effectively reduce the escalation of IPF-LFs and IPF-ACEs senescence and the production of IL-6, an archetypal SASP cytokine. Thus, it has been demonstrated that damaged self-DNA stimulates cGAS, causing the senescence of fibroblast and the persistent of ageing in IPF and other fibrotic lung diseases (26, 27).

**Figure 6:**
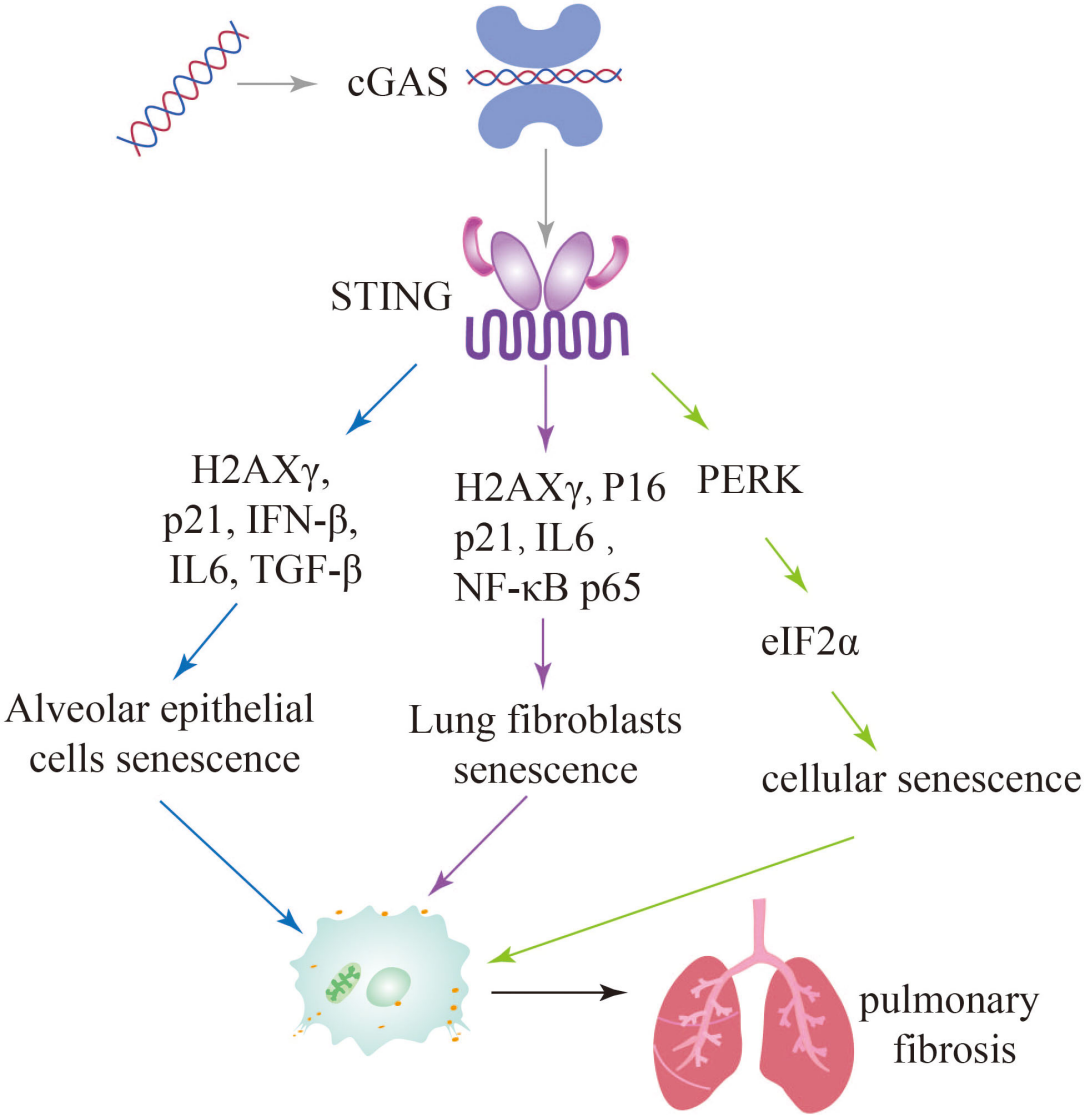
Activation of the cGAS-STING signaling pathway leads to further exacerbation of pulmonary fibrosis through multiple pathways of cellular senescence.

Zhang et al. identified a distinct signaling pathway involving STING-PKR-like endoplasmic reticulum kinase (PERK)-eI2α pathway, which differs from the conventional STING-TBK1-IRF3 pathway. This STING-PERK-eIF2α pathway was found to be involved in the initiation of cellular senescence mediated by cGAS-STING activation. In contrast, injury-induced apoptosis appears to be dependent on the STING-TBK1-IRF3 axis. The study further demonstrated that attenuating STING signaling through methods such as STING knockout (KO) mice, PERK KO mice, or the use of the STING inhibitor DMXAA, resulted in a reduction in lung fibrosis. These findings suggest that targeting the STING pathway may hold therapeutic potential for mitigating lung fibrosis (109). Since cGAS-STING pathway plays important roles in pulmonary fibrosis, these studies further suggest that inhibition of this pathway attenuates cellular senescence and further alleviates pulmonary fibrosis (**Figure 6**).

### 6.4 Exposure to silica particles and nanomaterial induced pulmonary fibrosis by activation of STING signaling

The lungs are the organs that directly connect the internal organs to the outside world. Various microparticles and fibers in the air enter the respiratory system and cause inflammation and fibrosis in the lungs. Silica (110, 111) and carbon nanotubes (CNTs) (112, 113) are versatile chemical raw materials that have been showed amazing potential in many fields. However, some carbon nanotube materials and silica particles are expected to pose increased risks to humans, identifying and managing their potential health problems is a major challenge. Silica causes silicosis, which is characterized by persistent lung inflammation and irreversible fibrosis (114–116). Multi-walled carbon nanotubes (MWCNTs) also cause lung inflammation and fibrosis (117, 118). However, until now, the underlying pathogenesis of which has not been clearly and fully elucidated.

Exposure to silica particles can trigger the release of pro-inflammatory and pro-fibrotic factors, such as IL-6, TNF-α, and TGF-β, which contribute to the acceleration of lung inflammation and fibrosis. This process involves the activation of the STING signaling pathway. Studies have shown that silencing STING can hinder the activation of M2 and M1 macrophages, as well as reduce the levels of fibrotic proteins such as Fn, Col-1, and α-SMA (30). Benmerzoug et al. found that respiratory exposure to silica in mice leads to the release of mitochondrial self-dsdna, which triggers the type I IFN pathway and induces cell death in the lungs. Activation of both STING and NLRP3 pathways was observed after silica exposure, leading to a cell death program and the release of pro-inflammatory cytokines. This process contributes to necrosis and apoptosis in a STING-dependent manner. Similar findings were also observed in silica-induced silicosis patients, where increased self-dsDNA release and upregulation of CXCL10 were observed (28). These studies highlight the role of the STING pathway in exacerbating lung fibrosis and inflammation caused by silica exposure. Targeting the STING signaling pathway may hold therapeutic potential in preventing silica-related silicosis and pulmonary fibrosis.

**Figure 7:**
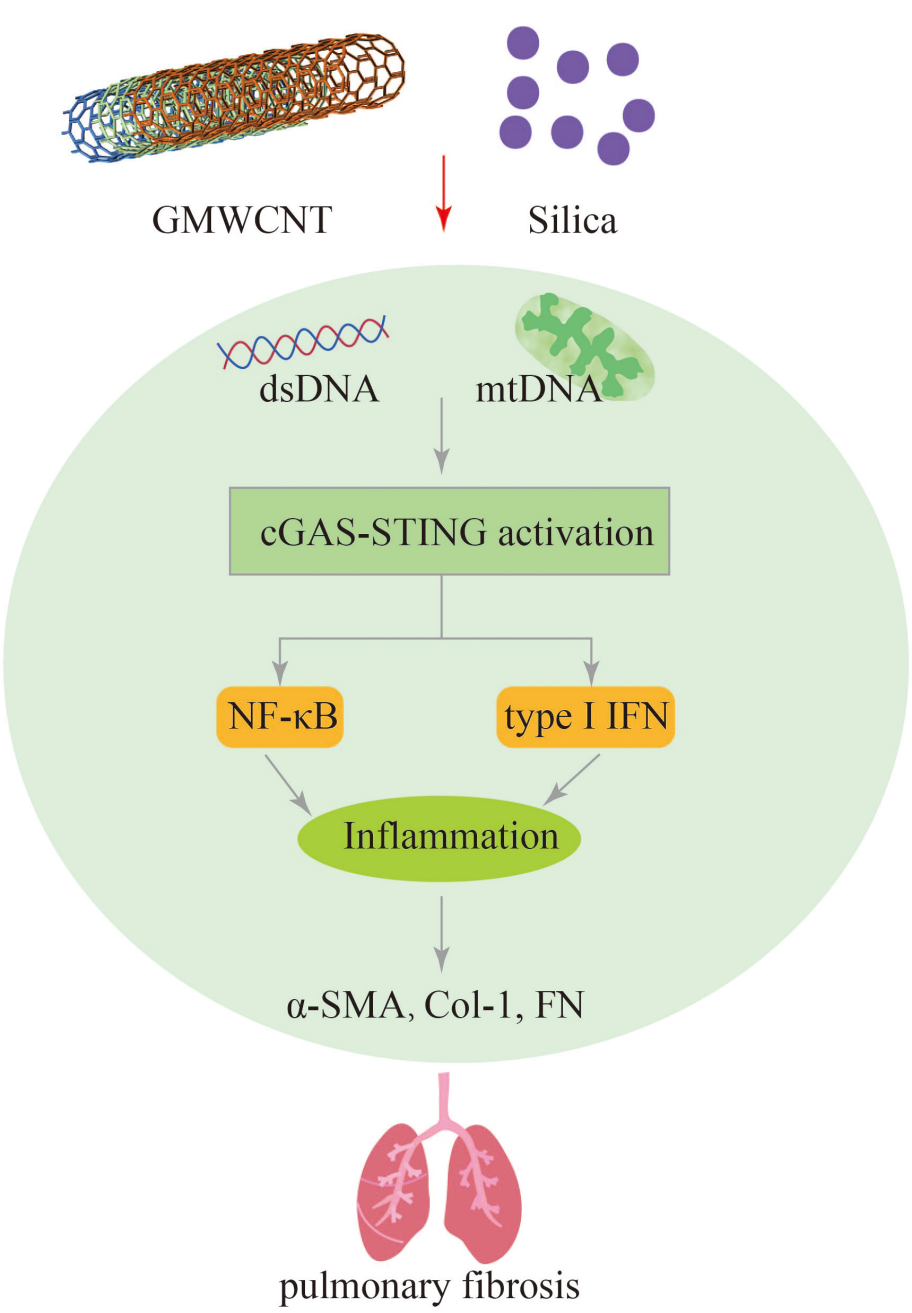
GMWCNT and silica induces pulmonary inflammation and fibrosis via activation of the cGAS-STING signaling pathway.

Exposure to GMWCNTs has been associated with lung fibrosis and inflammation. Activation of cGAS-STING signaling pathway, as evidenced by the increased expression of cGAS, STING, NF-κB, IL-1β, IFN-I TGF-β1, and collagen type I, has been observed in GMWCNT-induced lung injury. Treatment with a STING inhibitor (C176) has shown promising results in reducing mRNA and protein levels of certain cytokines, thereby alleviating pulmonary fibrosis (29). Therefore, targeting the STING signaling pathway could be a potential therapeutic strategy for mitigating lung inflammation and fibrosis induced by exposure to silica, GMWCNTs and other harmful microparticles and fibers (**Figure 7**).

## 7 Conclusions and perspectives

As an innate immune pathway, the role of cGAS-STING pathway in immune-related diseases is self-evident (74, 119). Despite, cGAS being discovered only in 2013 (39), the cGAS- STING pathway has progressed very rapidly in terms of its role in host defense and inflammation (41, 120). There is now a wealth of evidence showing that the cGAS-STING pathway plays an important role in lung disease (23), and a growing body of data suggests that the pathway is also active in pulmonary fibrosis. The JAK/STAT pathway plays a critical role in immune-inflammatory regulation. However, its extensive crosstalk with multiple pathways, such as MAPK pathway, Notch pathway, and PI3K/AKT/mTOR pathway, raises concerns about the specificity of targeting this pathway and the potential for side effects (121, 122). On the other hand, the cGAS-STING pathway specifically plays a role in recognizing DNA and activating innate immune responses. Targeting the cGAS-STING pathway offers the potential for more specific and selective modulation of immune responses, particularly focusing on DNA-induced inflammatory and fibrotic processes in lung fibrosis. Type I IFN serves as a crucial downstream effector of STING signaling, and the inhibition of cGAS or STING has shown effectiveness in suppressing inflammation. Several inhibitors of cGAS and STING are currently in preclinical development. The development of cGAS and STING-related drugs holds a promising future.

Pirfenidone and nintedanib are currently FDA-approved drugs for the treatment of lung fibrosis. However, their therapeutic mechanisms are known to be multifaceted. The anti-inflammatory effects of nintedanib and pirfenidone have been well-documented (123, 124). Studies have indicated elevated levels of mtDNA in the alveolar lavage fluid of patients with lung fibrosis (125). Lung injury-induced reactive oxygen species (ROS) release can lead to the release of mtDNA (126, 127). Notably, nintedanib and pirfenidone possess antioxidant properties (128, 129, 130), suggesting that they may exert therapeutic effects by inhibiting mtDNA release, preventing DNA binding to cGAS, and subsequently inhibiting the cGAS-STING pathway and its downstream inflammatory pathways.

In this review, we provided an overview of the current understanding of the cGAS-STING signaling pathway in pulmonary fibrosis and discussed its potential as therapeutic targets. The cGAS-STING pathway is activated in pulmonary fibrosis, and its activation of this pathway further aggravates the development of pulmonary fibrosis. Targeting the cGAS-STING pathway is of great significance in the treatment of pulmonary fibrosis, and many studies have shown that both natural products and small molecule inhibitors are effective in the management of lung fibrosis. In contrast, lung fibrosis caused by STING mutations remains an urgent area of investigation that requires further research. In summary, targeting the cGAS-STING signaling pathway holds a significant potential direction for therapeutic intervention in pulmonary fibrosis.

## Author contributions

JZ: Conceptualization, Project administration, Visualization, Writing – original draft, Writing – review & editing. LZ: Writing – review & editing. YC: Writing – review & editing. XF: Writing – review & editing. BL: Writing – review & editing. CM: Conceptualization, Funding acquisition, Project administration, Resources, Supervision, Visualization, Writing – original draft, Writing – review & editing.
