# Supplementary material for: The role of cGAS-STING signaling in pulmonary fibrosis and its therapeutic potential
Source: Front Immunol. 2023 Oct 25;14:1273248. doi: 10.3389/fimmu.2023.1273248 (PMC10642193; doi:10.3389/fimmu.2023.1273248)
Supplement: Supplementary file 1 [file DataSheet_1.docx]

# Overview of cGAS and STING agonists

The activation of the cGAS-STING pathway has shown promise in suppressing tumors and other diseases by enhancing tumor immune surveillance through mechanisms such as accelerated cancer cell senescence and cell death. Therefore, cGAS-STING agonists have been employed for the treatment of various diseases. (**Table S1**).

# cGAS activator

It is well known that since the discovery of cGAS, DNA has acted as the sole activator of cGAS. However, in 2020, Zhao et al reported that Mn^2+^-activated cGAS undergoes a conformational change. This conformational change results in the formation of a unique η1 helix, which plays an important role in allowing substrate entry and facilitating the synthesis of 2'3-cGAMP (Zhao et al. 2020).

# STING-nucleotidic agonists

Cyclic dinucleotides (CDNs) are important messenger molecules in nature. The role of the second messenger molecule AMP and cGMP has been intensively studied, with many different versions of c-GMP-AMP (cGAMP) found in bacterial and mammalian cells (Danilchanka and Mekalanos 2013). The family of cyclic AMP-GMP molecules includes various subtypes, including 3′3’-cGAMP, 2′3’-cGAMP, 3′5’-cGAMP, and 2′5’-cGAMP (Gao et al. 2013). When dsDNA is present, cGAS enzymatically catalyzes the generation of cGAMP from ATP and GTP. 2′3'-cGAMP binds to STING and activates a downstream pathway that triggers the activation of the type I IFN reaction (Sun et al. 2013).

Burdette et al. found that STING can be used as a cyclic dinucleotide sensor and function as a DNA sensor. The direct binding of to cyclic di-GMP (c-di-GMP) induces a STING-dependent type I interferon response (Burdette et al. 2011). Notably, 2′3′-cGAMP and 3′3’-cGAMP induce conformational rearrangements in STING and are potent inducer of IFNβ in mammalian cells (Zhang et al. 2013, Gao et al. 2015). The synthesis of hybrid disulfide-linked CDNs (ML RR-S2 CDA and ML RR-S2 CDG) has been shown to efficiently induce STING-dependent signaling in mouse and human immune cells. This activation leads to the stimulation of an effective systemic CD8+ T-cell immune response, which holds potential for antitumor immunity (Corrales et al. 2015).

# STING-non-CDN agonists

Emerging evidences indicate that non-CDN drugs play a critical role in activating the cGAS-STING pathway of antitumour immunity. 5,6-Dimethylflavone-4-acetic acid (DMXAA) directly binds to STING, activates the STING signaling pathway and induces the generation of inflammatory cytokines (Prantner et al. 2012). A linkage strategy was utilized in order to synergise two symmetrically related aminobenzimidazole (ABZI) analogues, thereby creating linked ABZIs (diABZIs) with stronger binding to STING and greater cellular functionality, which act as STING agonists to stimulate potent anti-tumour activity (Ramanjulu et al. 2018). Another compound, α-mangostin, binds to human STING and activates it, leading to production of type I interferons (Zhang et al. 2018). Unlike cGAMP, some small-molecule non-nucleotide STING agonists (CF501, CMA, DSDP) have the advantage of easier to triggers the STING pathway (Liu et al. 2022, Cavlar et al. 2013, Liu et al. 2017).

**Table S1. cGAS and STING agonists**

| **cGAS and STING agonists** | | **References** |
| --- | --- | --- |
| *cGAS agonist* | DNA | (Sun et al. 2013) |
|  | Mn2+ | (Zhao et al. 2020) |
| *STING-nucleotidic agonists* | Cyclic di-GMP | (Burdette et al. 2011) |
|  | 2′3′-cGAMP | (Zhang et al. 2013) |
|  | 3′3′-cGAMP | (Martin et al. 2017) |
|  | ML-RR-S2-CDG | (Corrales et al. 2015) |
|  | ML-RR-S2 CDA | (Corrales et al. 2015) |
| *STING-non-CDN agonists* | DMXAA | (Prantner et al. 2012) |
|  | DiABZI | (Ramanjulu et al. 2018) |
|  | α-mangostin | (Zhang et al. 2018) |
|  | CF501 | (Liu et al. 2022) |
|  | CMA | (Cavlar et al. 2013) |
|  | DSDP | (Liu et al. 2017) |

# References

Burdette, D. L., K. M. Monroe, K. Sotelo-Troha, J. S. Iwig, B. Eckert, M. Hyodo, Y. Hayakawa, and R. E. Vance. 2011. "STING is a direct innate immune sensor of cyclic di-GMP." *Nature* 478 (7370):515-8. doi: 10.1038/nature10429.

Cavlar, T., T. Deimling, A. Ablasser, K. P. Hopfner, and V. Hornung. 2013. "Species-specific detection of the antiviral small-molecule compound CMA by STING." *EMBO J* 32 (10):1440-50. doi: 10.1038/emboj.2013.86.

Corrales, L., L. H. Glickman, S. M. McWhirter, D. B. Kanne, K. E. Sivick, G. E. Katibah, S. R. Woo, E. Lemmens, T. Banda, J. J. Leong, K. Metchette, T. W. Dubensky, Jr., and T. F. Gajewski. 2015. "Direct Activation of STING in the Tumor Microenvironment Leads to Potent and Systemic Tumor Regression and Immunity." *Cell Rep* 11 (7):1018-30. doi: 10.1016/j.celrep.2015.04.031.

Danilchanka, O., and J. J. Mekalanos. 2013. "Cyclic dinucleotides and the innate immune response." *Cell* 154 (5):962-970. doi: 10.1016/j.cell.2013.08.014.

Gao, J., J. Tao, W. Liang, M. Zhao, X. Du, S. Cui, H. Duan, B. Kan, X. Su, and Z. Jiang. 2015. "Identification and characterization of phosphodiesterases that specifically degrade 3'3'-cyclic GMP-AMP." *Cell Res* 25 (5):539-50. doi: 10.1038/cr.2015.40.

Gao, P., M. Ascano, Y. Wu, W. Barchet, B. L. Gaffney, T. Zillinger, A. A. Serganov, Y. Liu, R. A. Jones, G. Hartmann, T. Tuschl, and D. J. Patel. 2013. "Cyclic [G(2',5')pA(3',5')p] is the metazoan second messenger produced by DNA-activated cyclic GMP-AMP synthase." *Cell* 153 (5):1094-107. doi: 10.1016/j.cell.2013.04.046.

Liu, B., L. Tang, X. Zhang, J. Ma, M. Sehgal, J. Cheng, X. Zhang, Y. Zhou, Y. Du, J. Kulp, J. T. Guo, and J. Chang. 2017. "A cell-based high throughput screening assay for the discovery of cGAS-STING pathway agonists." *Antiviral Res* 147:37-46. doi: 10.1016/j.antiviral.2017.10.001.

Liu, Z., J. Zhou, W. Xu, W. Deng, Y. Wang, M. Wang, Q. Wang, M. Hsieh, J. Dong, X. Wang, W. Huang, L. Xing, M. He, C. Tao, Y. Xie, Y. Zhang, Y. Wang, J. Zhao, Z. Yuan, C. Qin, S. Jiang, and L. Lu. 2022. "A novel STING agonist-adjuvanted pan-sarbecovirus vaccine elicits potent and durable neutralizing antibody and T cell responses in mice, rabbits and NHPs." *Cell Res* 32 (3):269-287. doi: 10.1038/s41422-022-00612-2.

Martin, T. L., J. Jee, E. Kim, H. E. Steiner, E. Cormet-Boyaka, and P. N. Boyaka. 2017. "Sublingual targeting of STING with 3'3'-cGAMP promotes systemic and mucosal immunity against anthrax toxins." *Vaccine* 35 (18):2511-2519. doi: 10.1016/j.vaccine.2017.02.064.

Prantner, D., D. J. Perkins, W. Lai, M. S. Williams, S. Sharma, K. A. Fitzgerald, and S. N. Vogel. 2012. "5,6-Dimethylxanthenone-4-acetic acid (DMXAA) activates stimulator of interferon gene (STING)-dependent innate immune pathways and is regulated by mitochondrial membrane potential." *J Biol Chem* 287 (47):39776-88. doi: 10.1074/jbc.M112.382986.

Ramanjulu, J. M., G. S. Pesiridis, J. Yang, N. Concha, R. Singhaus, S. Y. Zhang, J. L. Tran, P. Moore, S. Lehmann, H. C. Eberl, M. Muelbaier, J. L. Schneck, J. Clemens, M. Adam, J. Mehlmann, J. Romano, A. Morales, J. Kang, L. Leister, T. L. Graybill, A. K. Charnley, G. Ye, N. Nevins, K. Behnia, A. I. Wolf, V. Kasparcova, K. Nurse, L. Wang, A. C. Puhl, Y. Li, M. Klein, C. B. Hopson, J. Guss, M. Bantscheff, G. Bergamini, M. A. Reilly, Y. Lian, K. J. Duffy, J. Adams, K. P. Foley, P. J. Gough, R. W. Marquis, J. Smothers, A. Hoos, and J. Bertin. 2018. "Design of amidobenzimidazole STING receptor agonists with systemic activity." *Nature* 564 (7736):439-443. doi: 10.1038/s41586-018-0705-y.

Sun, L., J. Wu, F. Du, X. Chen, and Z. J. Chen. 2013. "Cyclic GMP-AMP synthase is a cytosolic DNA sensor that activates the type I interferon pathway." *Science* 339 (6121):786-91. doi: 10.1126/science.1232458.

Zhang, X., H. Shi, J. Wu, X. Zhang, L. Sun, C. Chen, and Z. J. Chen. 2013. "Cyclic GMP-AMP containing mixed phosphodiester linkages is an endogenous high-affinity ligand for STING." *Mol Cell* 51 (2):226-35. doi: 10.1016/j.molcel.2013.05.022.

Zhang, Y., Z. Sun, J. Pei, Q. Luo, X. Zeng, Q. Li, Z. Yang, and J. Quan. 2018. "Identification of alpha-Mangostin as an Agonist of Human STING." *ChemMedChem* 13 (19):2057-2064. doi: 10.1002/cmdc.201800481.

Zhao, Z., Z. Ma, B. Wang, Y. Guan, X. D. Su, and Z. Jiang. 2020. "Mn(2+) Directly Activates cGAS and Structural Analysis Suggests Mn(2+) Induces a Noncanonical Catalytic Synthesis of 2'3'-cGAMP." *Cell Rep* 32 (7):108053. doi: 10.1016/j.celrep.2020.108053.
